# Development and internal validation of a prediction model for early anastomotic leak after minimally invasive esophagectomy: a prospective cohort study

**DOI:** 10.1080/07853890.2026.2726636

**Published:** 2026-09-12

**Authors:** Zhi-Bo Feng, Xuan-Feng Ren, Meng-Hua Xue, Pei-Long Bao, He Xu, Fan Gao, Yao Ma, Jin-Bo Zhao, Yu-Quan Bai

**Affiliations:** ^a^ Department of Thoracic Surgery, Tangdu Hospital, The Fourth Military Medical University, Xi’an, China; ^b^ School of Medicine, Northwest University, Xi’an, China

**Keywords:** Esophageal cancer, early postoperative endoscopy, anastomotic leak, gastric conduit ischemic length, postoperative serum albumin

## Abstract

**Background:**

Anastomotic leak (AL) remains a complication after minimally invasive esophagectomy (MIE), affecting 10–20% of patients and increasing morbidity and mortality. Although local conduit perfusion and systemic nutritional status are central to anastomotic healing, no validated bedside tool integrates quantitative early endoscopic findings with systemic indicators to predict postoperative AL.

**Methods:**

This prospective single-center cohort study enrolled 583 patients who underwent MIE at Tangdu Hospital. Standardized endoscopy within 72 h quantified gastric conduit ischemic length, and postoperative serum albumin was measured. Multivariable logistic regression and receiver operating characteristic (ROC) analysis identified independent predictors and optimal cutoffs to develop a risk-stratification system. A Lasso-regularized logistic regression (Lasso LR) model was developed and internally validated (7:3 split), with performance assessed by area under the curve (AUC), calibration curves, and decision curve analysis. Associations with ECCG-defined AL severity were also assessed.

**Results:**

Gastric conduit ischemic length ≥3.5 cm (OR 3.361, *p* = 0.043) and postoperative serum albumin <31.75 g/L (OR 0.886, *p* < 0.001) were independent predictors. The Lasso LR model achieved an internal validation AUC of 0.727 (95% CI: 0.641–0.810). The risk-stratification system yielded AL rates of 11.00%, 22.73%, and 73.68% across low-, medium-, and high-risk groups (*p* < 0.001). Both predictors correlated with ECCG severity. A nomogram and web-based calculators were developed for bedside use.

**Conclusion:**

Quantitative gastric conduit ischemic length and postoperative serum albumin enabled individualized AL risk assessment within 72 h after MIE. By integrating local perfusion and systemic healing capacity, the model and digital tools provide a framework for early targeted postoperative management.

**Trial registration:**

Chinese Clinical Trial Registry, ChiCTR2100054550. Registered December 19, 2021, https://www.chictr.org.cn/.

## Introduction

Esophageal cancer is a highly aggressive malignancy of the digestive tract associated with a poor prognosis. Its global burden is substantial, with particularly high incidence in China, which accounts for approximately half of all cases worldwide [1]. This burden also varies by sex and region; males and rural patients often present at more advanced stages and experience worse outcomes. Whether such disparities affect postoperative risk prediction is unclear. Surgery remains the cornerstone of curative treatment. However, anastomotic leak (AL) remains a major postoperative complication with an incidence of 10%–20%, significantly impairing postoperative recovery and long-term quality of life [2,3]. The occurrence of AL is associated with markedly increased mortality, prolonged hospital stays, and substantially higher healthcare costs [4,5]. Consequently, there is an urgent clinical need to establish effective strategies for early warning and intervention. However, a critical translational gap persists between basic research findings and clinical practice: despite growing evidence linking anastomotic perfusion and nutritional status to healing outcomes, no validated tool currently exists to convert these insights into objective, quantifiable bedside decision criteria applicable within the early postoperative period.

The pathophysiology of AL is complex and involves multiple interacting factors. Adequate local blood supply to the anastomosis has long been regarded as a cornerstone of healing [6]. However, intraoperative assessment of distal gastric conduit perfusion often relies on the surgeon’s subjective judgment, lacking objective and quantifiable measures. At the same time, systemic patient factors are equally critical to anastomotic healing [7,8]. Studies have shown that systemic metabolic alterations following surgical stress, such as postoperative hypoalbuminemia, are closely associated with postoperative complications. These alterations may impair anastomotic healing by affecting tissue edema, immune function, and collagen synthesis [9]. In essence, anastomotic healing is not an isolated local event but rather the result of an interaction between the local microenvironment and systemic metabolic status. Nonetheless, whether combining objective local indicators with systemic markers provides additional value for the early prediction of AL remains unclear.

Early postoperative endoscopy offers a unique opportunity for direct evaluation of the anastomosis during the initial phase of healing. Our team’s previous retrospective work demonstrated that endoscopic assessment is safe and does not increase complication risks when performed within the critical 72-hour window for clinical intervention following minimally invasive esophagectomy (MIE) [10]. However, current interpretation of endoscopic findings at the anastomosis remains largely qualitative [11,12]. In the context of early postoperative assessment, how to convert qualitative endoscopic features into quantitative risk indicators and integrate them with systemic parameters to construct a robust predictive tool remains a critical blind spot in current research. High-quality prospective data are needed to establish and validate the reliability of such quantitative criteria.

To address this gap, a prospective cohort study was conducted in which quantitative endoscopic features and systemic indicators were systematically assessed within 72 h after MIE. The primary objective of this study was to identify independent predictors of AL from these early assessments and to develop and internally validate an individualized risk-prediction tool for AL in patients undergoing MIE. This tool is intended for use by thoracic surgeons at the bedside within 72 h postoperatively to stratify patients into risk groups and inform the intensity of postoperative monitoring and early intervention. The novelty of this study lies in combining endoscopically quantified gastric conduit ischemia, which directly reflects local conduit perfusion and ischemic injury at the anastomotic site, with postoperative serum albumin, an accessible indicator of the systemic metabolic and inflammatory conditions relevant to tissue repair. In contrast to previously published prediction models, which have largely relied on conventional clinical and laboratory variables [13,14], this strategy was designed to capture both the local biological substrate of anastomotic healing and the patient’s systemic healing capacity within the critical early postoperative window.

Based on the evidence outlined above, greater endoscopically quantified gastric conduit ischemic length and lower postoperative serum albumin were hypothesized to be associated with an increased risk of AL and to reflect complementary aspects of local conduit perfusion and systemic healing capacity. It was further hypothesized that both variables would retain independent predictive value when considered jointly. Accordingly, this study aimed to evaluate these associations and to develop and internally validate an individualized tool for early postoperative AL risk stratification after MIE.

## Methods

### Study design and participants

**Figure 1.**
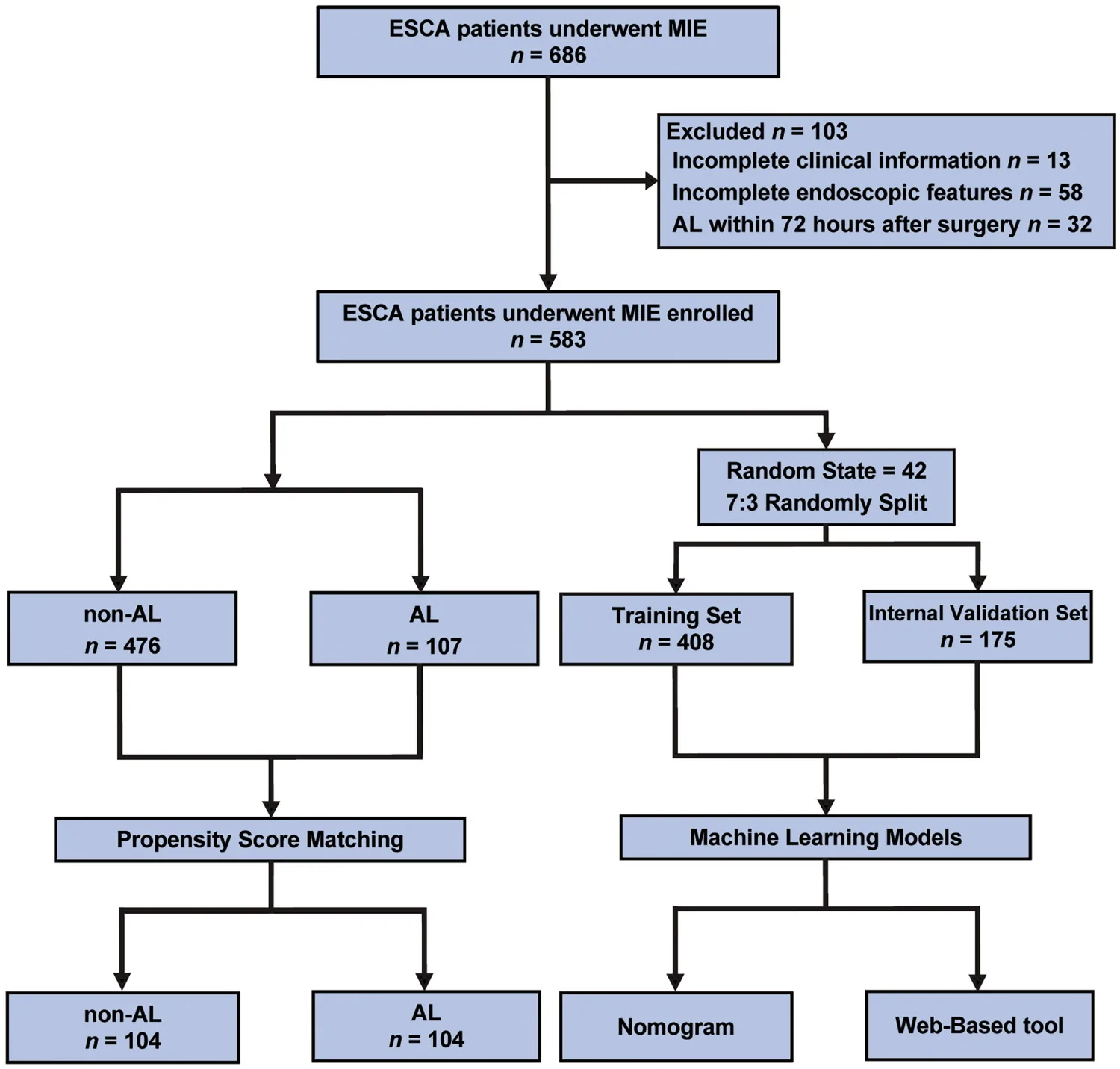
Flowchart of the overall study design. ESCA, esophageal cancer; MIE, minimally invasive esophagectomy; AL,  anastomotic leak.

This study was reported in accordance with the Transparent Reporting of a Multivariable Prediction Model for Individual Prognosis or Diagnosis, Extended for Artificial Intelligence (TRIPOD+AI) guidelines [15]. The completed checklist is provided in Supplementary Material 1. This single-center prospective cohort study was conducted in a tertiary-care inpatient setting at Tangdu Hospital, a high-volume MIE center in northwest China. Standardized surgical protocols and routine 72-hour endoscopy ensured uniform data collection. Patients with esophageal or esophagogastric junction (EGJ) cancer scheduled for MIE between August 3, 2021, and November 30, 2025, were prospectively enrolled. Eligible patients were required to have completed early endoscopy within 72 h postoperatively and to have complete clinical and endoscopic data available. Of 686 screened patients, 13 patients with incomplete clinical data, 58 with incomplete endoscopic data, and 32 who developed or were diagnosed with AL within 72 h postoperatively were excluded. Consequently, the final cohort comprised 583 patients, consisting of 476 without AL and 107 who subsequently developed AL (Figure 1).

### Inclusion and exclusion criteria

Patients were evaluated for eligibility prior to enrollment. The inclusion criteria were as follows: age between 18 and 75 years; pathologically confirmed esophageal or EGJ cancer; having undergone MIE with gastric conduit reconstruction, including either cervical or intrathoracic anastomosis; smooth postoperative recovery with stable vital signs and no requirement for mechanical ventilation; body mass index (BMI) between 18.0 and 28.0 kg/m^2^.

The exclusion criteria were as follows: severe respiratory dysfunction, defined as arterial partial pressure of oxygen (PaO_2_) < 60 mmHg, arterial partial pressure of carbon dioxide (PaCO_2_) > 45 mmHg, or forced expiratory volume in 1 s (FEV_1_) < 1000 mL; underlying severe comorbidities, including liver cirrhosis, myocardial infarction, or acute coronary syndrome; body weight loss > 15% within the preceding 6 months; synchronous multiple primary malignancies; upper margin of the tumor located < 5 cm from the upper esophageal sphincter; bilateral recurrent laryngeal nerve paralysis; and esophageal reconstruction using colon or jejunum interposition.

### Ethics approval and trial registration

The study was conducted in accordance with the Declaration of Helsinki and was approved by the Independent Ethics Committee of Tangdu Hospital, Fourth Military Medical University (approval number: 202108e05). It was registered with the Chinese Clinical Trial Register (registration number: ChiCTR2100054550), and written informed consent was obtained from all enrolled patients before participation.

### Outcome

The primary outcome was AL, defined according to the Esophagectomy Complications Consensus Group (ECCG) system as a full-thickness gastrointestinal defect involving the esophagus, anastomosis, staple line, or conduit [16]. Severity was categorized according to ECCG severity criteria by intervention intensity: Type I required no change in standard postoperative management or was treated conservatively with pharmacotherapy and nutritional/dietary modification; Type II required interventional but non-surgical therapy, including endoscopic management, stenting, negative-pressure wound therapy, or image-guided drainage; and Type III required surgical reoperation. Diagnoses were established by attending thoracic surgeons based on objective ECCG criteria and confirmed radiologically (contrast swallow, CT, or endoscopy); no machine learning algorithm was used for outcome adjudication. Blinding of assessors to the predictor variables was not feasible because AL identification required full access to clinical data for treatment decisions. To mitigate potential detection bias, standardized ECCG criteria were strictly applied, and all outcome data were extracted from electronic medical records using a standardized data collection form.

### Definitions of baseline patient characteristics and post-MIE short-term outcomes

Complete baseline data for all enrolled patients were extracted from the hospital’s electronic medical record system, covering four key domains: demographic and comorbidity characteristics, clinicopathological features, surgical indicators, and postoperative outcomes and complications. Neoadjuvant therapy was defined as receipt of chemotherapy, radiotherapy, immunotherapy, or a combination thereof prior to surgery. Operation time was defined as the total duration from skin incision to skin closure. Intraoperative blood transfusion was defined as the administration of allogeneic blood products, including red blood cells or plasma, during the procedure. For postoperative outcomes and complications, preoperative serum albumin was defined as the concentration measured before surgery, postoperative serum albumin was defined as the concentration measured within 72 h after surgery, and hypoproteinemia was defined as a serum albumin level < 30 g/L during the postoperative period (excluding patients with a pre-existing diagnosis of AL). The difference in serum albumin was calculated by subtracting the postoperative level from the preoperative baseline. Other postoperative short-term outcomes (including upper gastrointestinal hemorrhage, pneumonia, arrhythmia, chylothorax, venous thromboembolism, and organ dysfunction), length of hospital stay, and in-hospital mortality were evaluated according to standard clinical criteria. Detailed definitions for all postoperative short-term outcomes are provided in Supplementary Method.

### Surgical procedures and gastric conduit reconstruction

Patients underwent McKeown, Ivor Lewis, or Sweet esophagectomy according to tumor location and clinical characteristics. In all patients, a standardized narrow gastric conduit approximately 4 cm in width was constructed along the greater curvature with preservation of the right gastroepiploic vascular arcade. The conduit was reconstructed through the posterior mediastinal route. Anastomosis was performed in the neck for the McKeown procedure and in the right or left thoracic cavity for the Ivor Lewis or Sweet procedure, respectively. Anastomoses were classified as hand-sewn, mechanical end-to-side, mechanical end-to-end, or mechanical side-to-side; circular or linear staplers were used for mechanical anastomoses.

### Early endoscopic assessment and feature quantification after MIE

All patients underwent standardized early postoperative endoscopic evaluation within 72 h after MIE. The procedure was performed under topical anesthesia using a transnasal ultrathin electronic gastroscope (outer diameter 5.5–5.9 mm). Systematic examination was carried out sequentially to assess the larynx, residual esophagus, anastomosis, and gastric conduit tissues. Airway and vocal cord function were evaluated before and after tracheal extubation to ensure safety. The examinations were performed by experienced endoscopists, and the acquired images were reviewed independently by three endoscopists who were blinded to patient outcomes.

**Figure 2.**
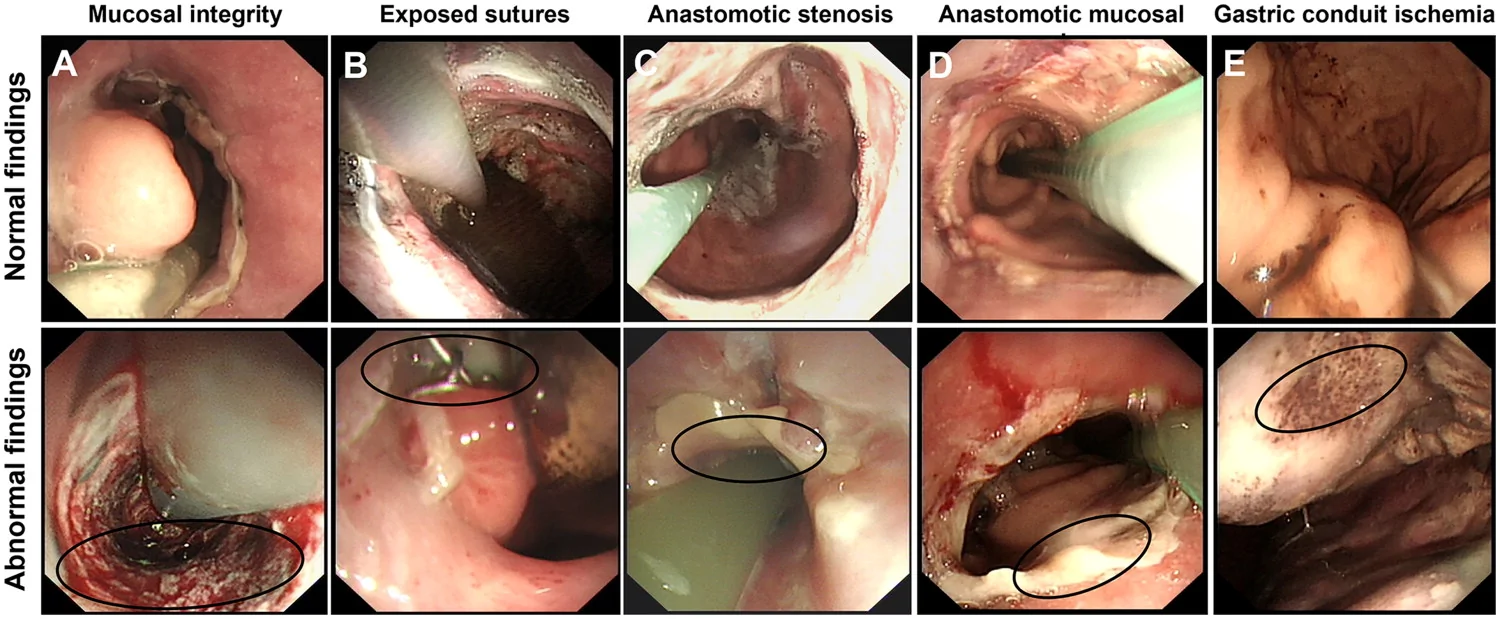
Normal (top) and corresponding abnormal findings (bottom) of early postoperative endoscopic examination. (A) Mucosal integrity; (B) Exposed sutures; (C) Anastomotic stenosis; (D) Anastomotic mucosal color; (E) Gastric conduit ischemia.

The blinded assessment focused on quantifying seven endoscopic features (Figure 2). Mucosal integrity: classified as intact or disrupted (defined by the presence of erosion, ulceration, or fissuring); suture exposure: graded according to the proportion of the anastomotic circumference involved (normal, < 1/4, 1/4 to < 1/2, 1/2 to < 3/4, or ≥ 3/4); anastomotic stricture: defined as an internal diameter ≤ 1 cm; anastomotic mucosal color and area of abnormal color: categorized as normal, white, gray-white, or black; the extent of involvement was graded using the same five-tier classification based on the proportion of the anastomotic circumference; gastric conduit mucosal color: categorized using the same classification as for the anastomosis; gastric conduit ischemia: defined as a composite finding of patchy or diffuse mucosal color changes, with or without submucosal ecchymosis or tissue rigidity [17]; gastric conduit ischemic length: for cases with confirmed ischemia, the maximum length of the ischemic area (cm) was measured along the long axis of the gastric conduit using calibrated software.

### Logistic regression analysis

To identify independent predictors of AL after MIE, univariate and multivariate logistic regression models were used to evaluate clinicopathological variables. Continuous variables were entered as raw values, while categorical variables were entered according to their predefined categories. Results are presented as odds ratios (ORs) with 95% confidence intervals (CIs). Variables with statistical significance (*p* < 0.05) in univariate analysis were included together in the subsequent multivariate logistic regression model for adjustment to determine the final independent predictors. Propensity score matching (PSM) was applied solely to balance baseline characteristics between the AL and non-AL groups and to confirm the robustness of group comparisons for postoperative outcomes and endoscopic features. The logistic regression analyses for identifying independent predictors of AL were conducted in the overall cohort (*n* = 583) to preserve statistical power and avoid the reduction in sample size inherent to matched analyses.

### Determination of optimal cutoff values and leak-free survival analysis

Receiver operating characteristic (ROC) curves were used to evaluate the predictive performance of continuous variables (gastric conduit ischemic length and postoperative serum albumin) for AL. Optimal cutoff values were determined by calculating the area under the curve (AUC) and the maximum Youden index (sensitivity + specificity − 1). The cutoff value for postoperative serum albumin was calculated using the overall cohort (*n* = 583). Because patients without ischemia were assigned an ischemic length of 0 cm and were automatically classified into the low-risk group, the optimal cutoff value for gastric conduit ischemic length, which was used to differentiate mild from severe ischemia, was calculated only in the subgroup of patients with confirmed ischemia (*n* = 106).

Based on the optimal cutoff values identified by ROC analysis, binary risk scores were assigned to the final independent predictors. The overall cohort (*n* = 583) was then stratified according to the total score into low-risk (0 points), medium-risk (1 point), and high-risk (2 points) groups. Postoperative leak-free survival curves were generated using the Kaplan-Meier method, with the endpoint defined as the time from surgery to radiologically or clinically confirmed AL. No post-discharge follow-up was performed; patients were observed during the index hospitalization, and those who did not develop AL were censored at hospital discharge. Differences in leak-free probability among the risk groups were compared using the log-rank test.

### Sample size

No formal a priori sample size calculation was performed. All eligible consecutive patients were enrolled during the predefined study period (August 3, 2021, to November 30, 2025) to maximize representativeness. The overall cohort (*n* = 583) was randomly divided into a training set (*n* = 408, 75 AL events) and an internal validation set (*n* = 175, 32 AL events) at a 7:3 ratio using a fixed random state of 42, with stratification by the outcome to preserve the event distribution. The training set provided an events-per-variable (EPV) of 37.5 (75 events/2 predictors), exceeding the recommended minimum of 10 events per predictor. The internal validation set contained 32 outcome events and yielded an AUC of 0.727 (95% CI: 0.641–0.810), supporting adequate sample size for reliable internal validation.

### Model development and internal validation

Seven candidate models were developed using the predictors identified by multivariable analysis: Lasso logistic regression (LR), simple logistic regression, logistic regression with interaction terms, polynomial logistic regression, random forest, gradient boosting, and support vector machine (SVM). These models were compared to determine whether nonlinear, interaction-based, or regularized approaches improved performance over a parsimonious logistic model. For Lasso logistic regression, the regularization parameter was selected by 10-fold cross-validation. Model discrimination was assessed using ROC-AUC with 95% CIs and precision–recall curves (PRCs) with average precision (AP) in the training and internal validation sets.

### Model interpretation and clinical utility assessment

Standardized regression coefficients were calculated to compare the relative contributions of the predictors in the final Lasso logistic regression model. The model was translated into a nomogram and an interactive web-based risk calculator. Calibration was evaluated using 1000 bootstrap resamples, and decision curve analysis (DCA) was performed to estimate net benefit across a range of threshold probabilities.

### Statistical analysis

PSM was performed using 1:1 nearest-neighbor matching based on the baseline covariates listed in Table 1. Covariate balance was evaluated using the absolute standardized mean difference (ASMD), with ASMD < 0.1 indicating adequate balance. To address potential class imbalance, comparisons of postoperative outcomes and early endoscopic features between the AL and non-AL groups were repeated after 1:1 PSM to assess whether the observed group differences remained consistent after balancing baseline covariates. Categorical variables were compared using the χ^2^ test or Fisher’s exact test, and continuous variables were compared using the independent-samples t test or Mann–Whitney U test, as appropriate. Differences among the Non-AL and ECCG Type I–III groups were evaluated using the Kruskal–Wallis test, and associations with ECCG type were assessed using Spearman correlation. Statistical analyses were performed using SPSS version 25.0, Python version 3.10 with scikit-learn, and R version 4.2.1 with the ‘rms’ package. All tests were two-sided, with *p* < 0.05 considered statistically significant.

**Table 1.** Comparison of baseline characteristics between the AL and non-AL groups before and after propensity score matching.

Characteristics, *n* (%)	Before PSM			After PSM		
	non-AL (*n* = 476)	AL (*n* = 107)	*p*	non-AL (*n* = 104)	AL (*n* = 104)	*p*
**Age (years), (median [IQR])**	63.00 [58.00, 69.00]	61.00 [57.50, 68.00]	0.240	62.00 [58.00, 67.25]	61.50 [58.00, 68.00]	0.966
**Gender**			0.048			1.000
female	104 (21.85)	15 (14.02)		14 (13.46)	15 (14.42)	
male	372 (78.15)	92 (85.98)		90 (86.54)	89 (85.58)	
**Smoking**			0.407			0.324
Never smoked	206 (43.28)	41 (38.32)		47 (45.19)	39 (37.50)	
Current or former smoker	270 (56.72)	66 (61.68)		57 (54.81)	65 (62.50)	
**Drinking**			0.749			0.351
Never drink	355 (74.58)	82 (76.64)		72 (69.23)	79 (75.96)	
Current or former drinker	121 (25.42)	25 (23.36)		32 (30.77)	25 (24.04)	
**Hypertension**			0.450			0.327
No	359 (75.42)	85 (79.44)		76 (73.08)	83 (79.81)	
Yes	117 (24.58)	22 (20.56)		28 (26.92)	21 (20.19)	
**Diabetes**			0.403			0.218
No	435 (91.39)	101 (94.39)		92 (88.46)	98 (94.23)	
Yes	41 (8.61)	6 (5.61)		12 (11.54)	6 (5.77)	
**Neoadjuvant therapy**			0.172			0.120
No	118 (24.79)	34 (31.78)		23 (22.12)	34 (32.69)	
Yes	358 (75.21)	73 (68.22)		81 (77.88)	70 (67.31)	
**Tumor location**			0.921			0.926
Upper	22 (4.62)	4 (3.74)		3 (2.88)	4 (3.85)	
Middle	222 (46.64)	50 (46.73)		50 (48.08)	50 (48.08)	
Lower	232 (48.74)	53 (49.53)		51 (49.04)	50 (48.08)	
**Anastomotic site**			0.327			0.559
Cervical	298 (62.61)	73 (68.22)		66 (63.46)	71 (68.27)	
Intrathoracic	178 (37.39)	34 (31.78)		38 (36.54)	33 (31.73)	
**cT stage**			0.051			0.213
T1	121 (25.42)	30 (28.04)		22 (21.15)	29 (27.88)	
T2	88 (18.49)	30 (28.04)		26 (25.00)	29 (27.88)	
T3	233 (48.95)	38 (35.51)		51 (49.04)	37 (35.58)	
T4	34 (7.14)	9 (8.41)		5 (4.81)	9 (8.65)	
**cN stage**			0.621			0.451
N0	277 (58.19)	68 (63.55)		68 (65.38)	66 (63.46)	
N1	129 (27.10)	28 (26.17)		24 (23.08)	27 (25.96)	
N2	60 (12.61)	9 (8.41)		12 (11.54)	9 (8.65)	
N3	10 (2.10)	2 (1.87)		0 (0.00)	2 (1.92)	
**cTNM stage**			0.492			0.597
I	115 (24.16)	30 (28.04)		22 (21.15)	29 (27.88)	
II	188 (39.50)	46 (42.99)		53 (50.96)	44 (42.31)	
III	136 (28.57)	23 (21.50)		22 (21.15)	23 (22.12)	
IV	37 (7.77)	8 (7.48)		7 (6.73)	8 (7.69)	
**Pathological type**			0.289			0.206
Squamous carcinoma	384 (80.67)	83 (77.57)		87 (83.65)	80 (76.92)	
Adenocarcinoma	8 (1.68)	4 (3.74)		0 (0.00)	4 (3.85)	
AEG	76 (15.97)	16 (14.95)		14 (13.46)	16 (15.38)	
Others	8 (1.68)	4 (3.74)		3 (2.88)	4 (3.85)	
**Surgical approach**			0.368			0.885
McKeown	314 (65.97)	71 (66.36)		69 (66.35)	69 (66.35)	
Sweet	75 (15.76)	12 (11.21)		14 (13.46)	12 (11.54)	
Ivor Lewis	87 (18.28)	24 (22.43)		21 (20.19)	23 (22.12)	
**Anastomotic method**			0.497			0.554
Hand-sewn	36 (7.56)	12 (11.21)		7 (6.73)	11 (10.58)	
Mechanical (end-to-side)	417 (87.61)	88 (82.24)		93 (89.42)	87 (83.65)	
Mechanical (end-to-end)	15 (3.15)	4 (3.74)		1 (0.96)	3 (2.88)	
Mechanical (side-to-side)	8 (1.68)	3 (2.80)		3 (2.88)	3 (2.88)	
**Operation time (min) (median [IQR])**	255.00 [215.00, 300.00]	265.00 [216.50, 301.00]	0.674	262.50 [220.00, 305.00]	265.00 [213.75, 300.00]	0.616
**Intraoperative blood transfusion**			0.152			0.459
No	448 (94.12)	96 (89.72)		97 (93.27)	93 (89.42)	
Yes	28 (5.88)	11 (10.28)		7 (6.73)	11 (10.58)	
**Number of lymph nodes dissected (median [IQR])**	22.00 [16.00, 30.00]	20.00 [13.00, 27.50]	0.029	21.00 [13.75, 27.25]	20.50 [13.00, 27.25]	0.879
**Number of metastatic lymph nodes (median [IQR])**	0.00 [0.00, 1.00]	0.00 [0.00, 0.50]	0.563	0.00 [0.00, 0.00]	0.00 [0.00, 0.25]	0.317

Values are n (%) unless otherwise indicated. PSM, propensity score matching; AL, anastomotic leak; AEG, adenocarcinoma of the esophagogastric junction; Others, esophageal small cell carcinoma, esophageal carcinosarcoma, esophageal neuroendocrine carcinoma and esophageal chondrosarcoma.

## Results

### Baseline characteristics of patients in the AL and non-AL groups before and after PSM

The overall cohort comprised 583 patients, including 107 patients in the AL group and 476 patients in the non-AL group. After 1:1 PSM (3 AL patients could not be matched due to caliper constraints), the PSM-matched cohort comprised 104 patients in each group (*n* = 208). Comparison of baseline characteristics (Table 1) showed significant between-group differences in sex (*p* = 0.048) and the number of dissected lymph nodes (*p* = 0.029) before PSM. After PSM, all included baseline covariates were well balanced between the two groups, with no statistically significant differences remaining (all *p* > 0.05). Moreover, the propensity-score distribution plot and covariate forest plot confirmed that the ASMDs for all variables were < 0.1 after matching (Figure S1). These findings indicate that the matched cohort provided improved baseline comparability for evaluating the association between AL and postoperative outcomes.

### Comparison of postoperative short-term outcomes between the AL and non-AL groups

To assess the association between AL and short-term postoperative outcomes, postoperative complications, serum albumin levels, and hospitalization-related parameters were compared between the two groups (Table 2). In the overall cohort (*n* = 583), patients in the AL group exhibited worse overall outcomes, with significantly higher incidences of hypoproteinemia, abnormal liver function, arrhythmia, and renal insufficiency, as well as longer hospital stays and higher in-hospital mortality (all *p* < 0.05).

**Table 2.** Comparison of postoperative short-term outcomes between the AL and non-AL groups before and after propensity score matching.

Events	Before PSM			After PSM		
	non-AL (*n* = 476)	AL (*n* = 107)	*p*	non-AL (*n* = 104)	AL (*n* = 104)	*p*
**Hypoproteinemia**			0.001*			0.012*
No	358 (75.21)	62 (57.94)		79 (75.96)	61 (58.65)	
Yes	118 (24.79)	45 (42.06)		25 (24.04)	43 (41.35)	
**Preoperative serum albumin (g/L), (median [IQR])**	43.70 [41.40, 45.80]	44.00 [41.00, 45.90]	0.863	44.05 [41.68, 46.00]	44.05 [41.10, 45.90]	0.396
**Postoperative serum albumin (g/L), (median [IQR])**	32.40 [30.08, 34.55]	30.40 [28.65, 33.25]	<0.001	32.40 [30.08, 34.58]	30.50 [28.70, 33.33]	**0.003**
**Difference serum albumin (g/L), (median [IQR])**	11.00 [8.88, 13.60]	12.30 [9.70, 15.45]	0.003	11.20 [9.18, 13.63]	12.20 [9.70, 15.25]	0.072
**Upper gastrointestinal hemorrhage**			0.499^#^			0.477^#^
No	473 (99.37)	105 (98.13)		104 (100.00)	102 (98.08)	
Yes	3 (0.63)	2 (1.87)		0 (0.00)	2 (1.92)	
**Abnormal liver function**			0.013*			0.124^#^
No	470 (98.74)	101 (94.39)		103 (99.04)	98 (94.23)	
Yes	6 (1.26)	6 (5.61)		1 (0.96)	6 (5.77)	
**Pneumonia**			0.216*			0.178*
No	384 (80.67)	80 (74.77)		86 (82.69)	77 (74.04)	
Yes	92 (19.33)	27 (25.23)		18 (17.31)	27 (25.96)	
**Arrhythmia**			0.016*			0.193*
No	457 (96.01)	96 (89.72)		99 (95.19)	93 (89.42)	
Yes	19 (3.99)	11 (10.28)		5 (4.81)	11 (10.58)	
**Chylothorax**			0.672^#^			1.000^#^
No	472 (99.16)	105 (98.13)		102 (98.08)	102 (98.08)	
Yes	4 (0.84)	2 (1.87)		2 (1.92)	2 (1.92)	
**Recurrent laryngeal nerve palsy**			0.839^#^			1.000^#^
No	458 (96.22)	104 (97.20)		102 (98.08)	101 (97.12)	
Yes	18 (3.78)	3 (2.80)		2 (1.92)	3 (2.88)	
**Renal insufficiency**			0.005^#^			0.442^#^
No	473 (99.37)	102 (95.33)		102 (98.08)	99 (95.19)	
Yes	3 (0.63)	5 (4.67)		2 (1.92)	5 (4.81)	
**Venous thromboembolism**			0.244^#^			1.000^#^
No	469 (98.53)	103 (96.26)		101 (97.12)	100 (96.15)	
Yes	7 (1.47)	4 (3.74)		3 (2.88)	4 (3.85)	
**Postoperative length of hospital stay (days), (median [IQR])**	12.00 [10.00, 14.00]	29.00 [19.50, 41.00]	<0.001	12.00 [10.00, 15.00]	29.00 [19.75, 41.00]	**<0.001**
**In-hospital mortality**			<0.001^#^			0.070^#^
Alive	476 (100.00)	102 (95.33)		104 (100.00)	99 (95.19)	
Dead	0 (0.00)	5 (4.67)		0 (0.00)	5 (4.81)	

Values are n (%) unless otherwise indicated. PSM, propensity score matching; AL, anastomotic leak. * Pearson χ² statistic. ^#^ Fisher exact χ² analysis.

To control for potential confounding bias, these outcomes were reassessed in the PSM-matched cohort (*n* = 208). The results demonstrated that the negative impact of AL on postoperative albumin metabolism and hospital stay duration was independent and robust. After matching, the incidence of postoperative hypoproteinemia remained significantly higher in the AL group than in the non-AL group (41.35% vs. 24.04%, *p* = 0.012), serum albumin levels measured within 72 h postoperatively were significantly lower [30.50 g/L (IQR: 28.70–33.33) vs. 32.40 g/L (IQR: 30.08–34.58), *p* = 0.003], and postoperative hospital stay was substantially prolonged [29.00 days (IQR: 19.75–41.00) vs. 12.00 days (IQR: 10.00–15.00), *p* < 0.001]. Notably, the between-group differences in abnormal liver function, arrhythmia, and renal insufficiency were no longer statistically significant after matching (all *p* > 0.05). Similarly, the difference in serum albumin levels between the AL and non-AL groups (*p* = 0.072) and the higher in-hospital mortality rate in the AL group (4.81% vs. 0.00%, *p* = 0.070) showed only borderline statistical significance after matching.

### Correlation between AL and early postoperative endoscopic features

To identify local endoscopic features associated with AL, seven quantitative indicators were compared between the two groups (Table 3). In the PSM-matched cohort (*n* = 208), which accounted for baseline confounding factors, only gastric conduit ischemic length (*p* = 0.006) and gastric conduit mucosal color (*p* = 0.017) differed significantly between the AL and non-AL groups. In contrast, the differences observed in the overall cohort for exposure sutures (*p* = 0.006) and anastomotic mucosal color area (*p* = 0.008) were no longer statistically significant after matching (*p* = 0.082 and *p* = 0.225). The remaining indicators, including mucosal integrity and anastomotic stenosis, were not significantly associated with AL either before or after matching.

**Table 3.** Comparison of early postoperative endoscopic findings between the AL and non-AL groups before and after propensity score matching.

Abnormal findings	Before PSM			After PSM		
	non-AL (*n* = 476)	AL (*n* = 107)	*p*	non-AL (*n* = 104)	AL (*n* = 104)	*p*
**Mucosal Integrity**			0.362^#^			0.364^#^
Yes	467 (98.11)	106 (99.07)		102 (98.08)	104 (100.00)	
No	9 (1.89)	1 (0.93)		2 (1.92)	0 (0.00)	
**Exposed sutures**			0.006^#^			0.082^#^
None	463 (97.27)	97 (90.65)		101 (97.12)	94 (90.38)	
Exposure <1/4	9 (1.89)	9 (8.41)		2 (1.92)	9 (8.65)	
1/4 < Exposure <2/4	1 (0.21)	1 (0.93)		0 (0.00)	1 (0.96)	
2/4 < Exposure <3/4	2 (0.42)	0 (0.00)		1 (0.96)	0 (0.00)	
Exposure >3/4	1 (0.21)	0 (0.00)		0 (0.00)	0 (0.00)	
**Anastomotic stenosis**			0.762^#^			1.000
Diameter >1cm	472 (99.16)	107 (100.00)		103 (99.04)	104 (100.00)	
Diameter ≤1cm	4 (0.84)	0 (0.00)		1 (0.96)	0 (0.00)	
**Anastomotic mucosal color area**			0.008*			0.225^#^
Normal	101 (21.22)	21 (19.63)		16 (15.38)	21 (20.19)	
Area <1/4	246 (51.68)	50 (46.73)		60 (57.69)	49 (47.12)	
1/4 < Area <2/4	73 (15.34)	11 (10.28)		14 (13.46)	11 (10.58)	
2/4 < Area <3/4	28 (5.88)	8 (7.48)		7 (6.73)	7 (6.73)	
Area >3/4	28 (5.88)	17 (15.89)		7 (6.73)	16 (15.38)	
**Anastomotic mucosal color**			0.247*			0.071*
Normal	115 (24.16)	22 (20.56)		20 (19.23)	22 (21.15)	
White	254 (53.36)	59 (55.14)		50 (48.08)	57 (54.81)	
Grayish-white	71 (14.92)	22 (20.56)		19 (18.27)	21 (20.19)	
Black	36 (7.56)	4 (3.74)		15 (14.42)	4 (3.85)	
**Gastric conduit ischemic length (cm), (median [IQR])**	0.00 [0.00, 0.00]	0.00 [0.00, 2.00]	<0.001	0.00 [0.00, 0.00]	0.00 [0.00, 2.00]	**0.006**
**Gastric conduit mucosal color**			<0.001*			**0.017** ^#^
Normal	408 (85.71)	69 (64.49)		87 (83.65)	67 (64.42)	
White	26 (5.46)	12 (11.21)		6 (5.77)	12 (11.54)	
Grayish-white	33 (6.93)	19 (17.76)		7 (6.73)	18 (17.31)	
Black	9 (1.89)	7 (6.54)		4 (3.85)	7 (6.73)	

Values in parentheses are percentages unless indicated others. EPE, early postoperative endoscopy; PSM, propensity score matching; AL, anastomotic leak. * Pearson χ² statistic. ^#^ Fisher exact χ² analysis.

### Identification of gastric conduit ischemic length and postoperative serum albumin as independent predictors of AL

**Figure 3.**
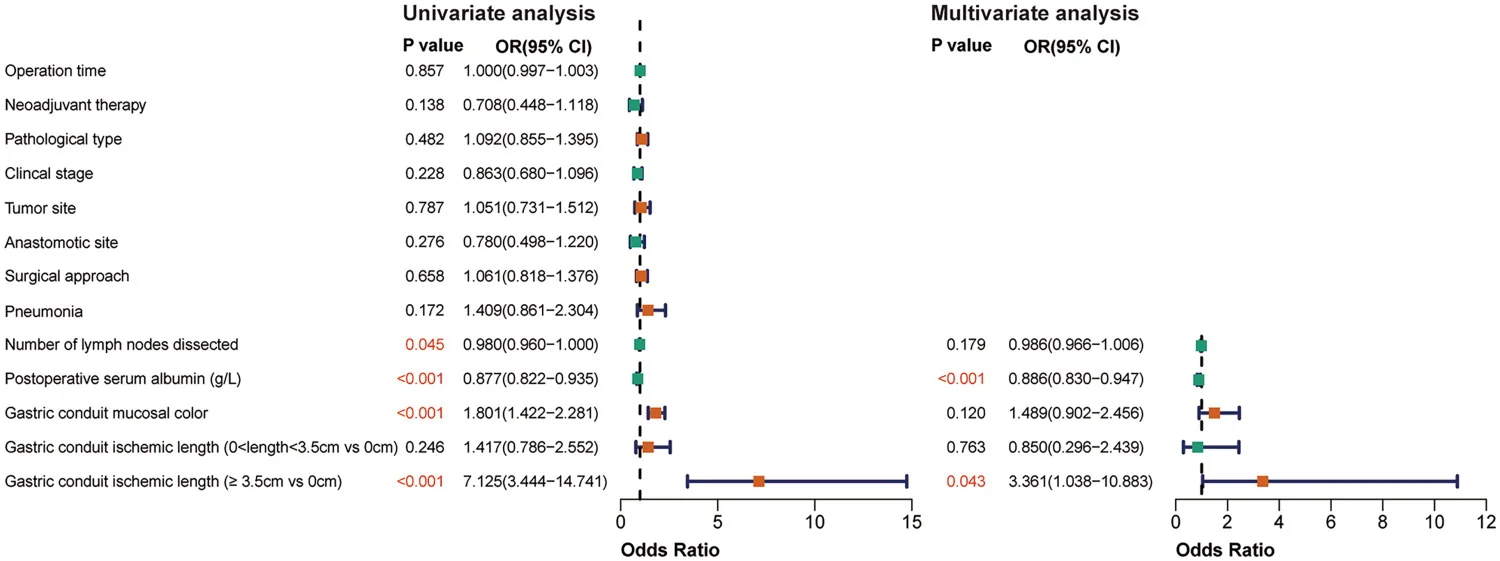
Forest plot of univariate and multivariate logistic regression analyses for predictors of AL after MIE. This figure presents the ORs with 95% CIs for the associations between candidate clinical variables and postoperative AL occurrence. In the univariate analysis, variables with statistically significant P values (*p* < 0.05) are highlighted in red. The multivariate logistic regression analysis further identified independent predictors of AL, with statistically significant *p* values (*p* < 0.05) also indicated in red.

**Figure 4.**
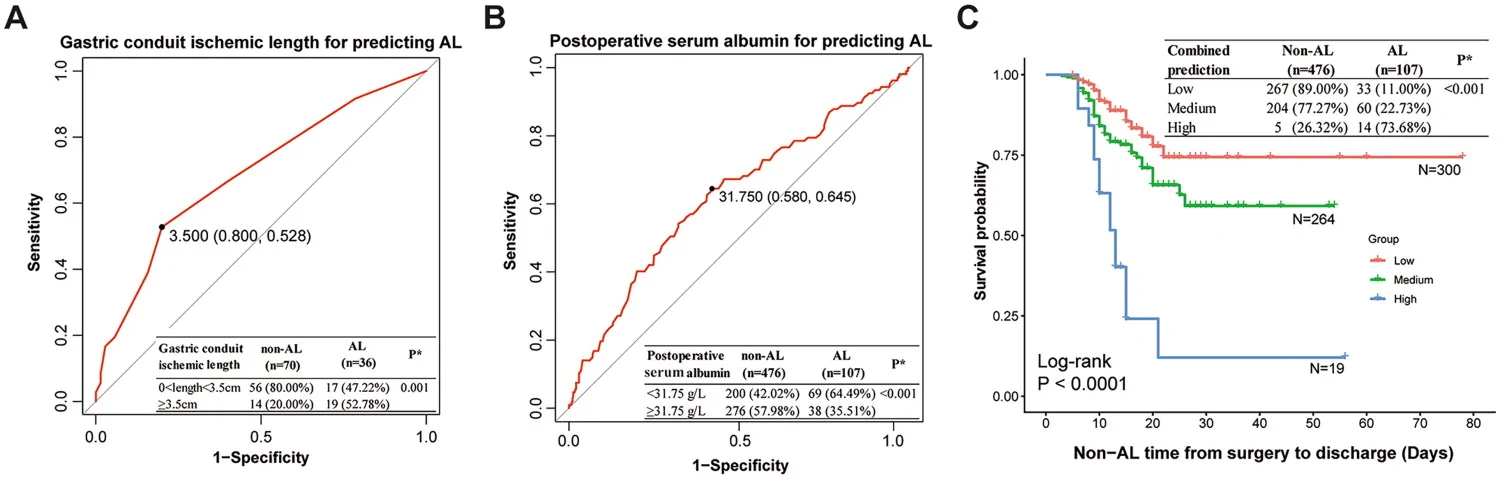
Determination of optimal cutoff values for predictors of AL after MIE and performance of the simple risk stratification model. (A) ROC curve for determining the optimal cutoff value of gastric conduit ischemic length for predicting AL. (B) ROC curve for determining the optimal cutoff value of postoperative serum albumin for predicting AL. (C) Kaplan-Meier curve of leak-free survival probability based on the simple risk stratification model. This model integrates two independent predictors: gastric conduit ischemic length (≥ 3.5 cm) and postoperative serum albumin (< 31.75 g/L). Patients were stratified into three risk groups according to their total score: low-risk (0 points), medium-risk (1 point), and high-risk (2 points). *p values were calculated using the Pearson chi-square test. AL, anastomotic leak.

To identify independent predictors of AL, univariable and multivariable logistic regression analyses were performed in the overall cohort of 583 patients (Figure 3). Given the distribution characteristics of gastric conduit ischemic length, ROC curve analysis was first conducted in patients with confirmed ischemia (*n* = 106) to convert this variable into a readily assessable categorical variable. The optimal cutoff for distinguishing mild from severe ischemia was 3.5 cm (specificity 80.00%, sensitivity 52.78%; Figure 4A). Subsequently, multiple clinical features previously reported in the literature [4,18–23], including this ischemic-length category, along with postoperative serum albumin and relevant endoscopic features, were entered into the logistic regression model. Univariate analysis showed that postoperative serum albumin (OR 0.877, 95% CI 0.822–0.935, *p* < 0.001), number of lymph nodes dissected (OR 0.980, 95% CI 0.960–1.000, *p* = 0.045), gastric conduit ischemic length (≥ 3.5 cm vs. 0 cm) (OR 7.125, 95% CI 3.444–14.741, *p* < 0.001), and gastric conduit mucosal color (OR 1.801, 95% CI 1.422–2.281, *p* < 0.001) were significantly associated with AL. After adjustment for potential confounders, multivariate analysis identified lower postoperative serum albumin (OR 0.886, 95% CI 0.830–0.947, *p* < 0.001) and gastric conduit ischemic length (≥ 3.5 cm vs. 0 cm; OR 3.361, 95% CI 1.038–10.883, *p* = 0.043) as two independent predictors of AL after MIE.

### Development and validation of a risk stratification model based on independent predictors

To convert postoperative serum albumin, the continuous independent predictor identified above, into an objective clinical criterion, ROC curve analysis was performed in the overall cohort (*n* = 583). The optimal cutoff value for predicting AL was 31.75 g/L (specificity 57.98%, sensitivity 64.49%; Figure 4B). Based on these two core classification criteria, a simple risk stratification model for postoperative AL was developed. Risk scores were assigned to the two independent predictors as follows: postoperative serum albumin ≥ 31.75 g/L was scored as 0 points, and < 31.75 g/L as 1 point; absence of gastric conduit ischemia (0 cm) or mild ischemia (0 cm < ischemic length < 3.5 cm) was scored as 0 points, and severe ischemia (≥ 3.5 cm) as 1 point. According to the total score, the overall cohort was stratified into three risk groups: low risk (0 points, *n* = 300), medium risk (1 point, *n* = 264), and high risk (2 points, *n* = 19). AL incidence increased significantly and stepwise with increasing risk score (11.00%, 22.73%, and 73.68%, respectively; *p* < 0.001; Figure 4C). These findings suggest that the simple stratification model effectively and intuitively discriminates patient populations at different risks of AL, providing a reference for early postoperative clinical intervention.

### Development and selection of predictive models within a multidimensional machine learning framework

**Figure 5.**
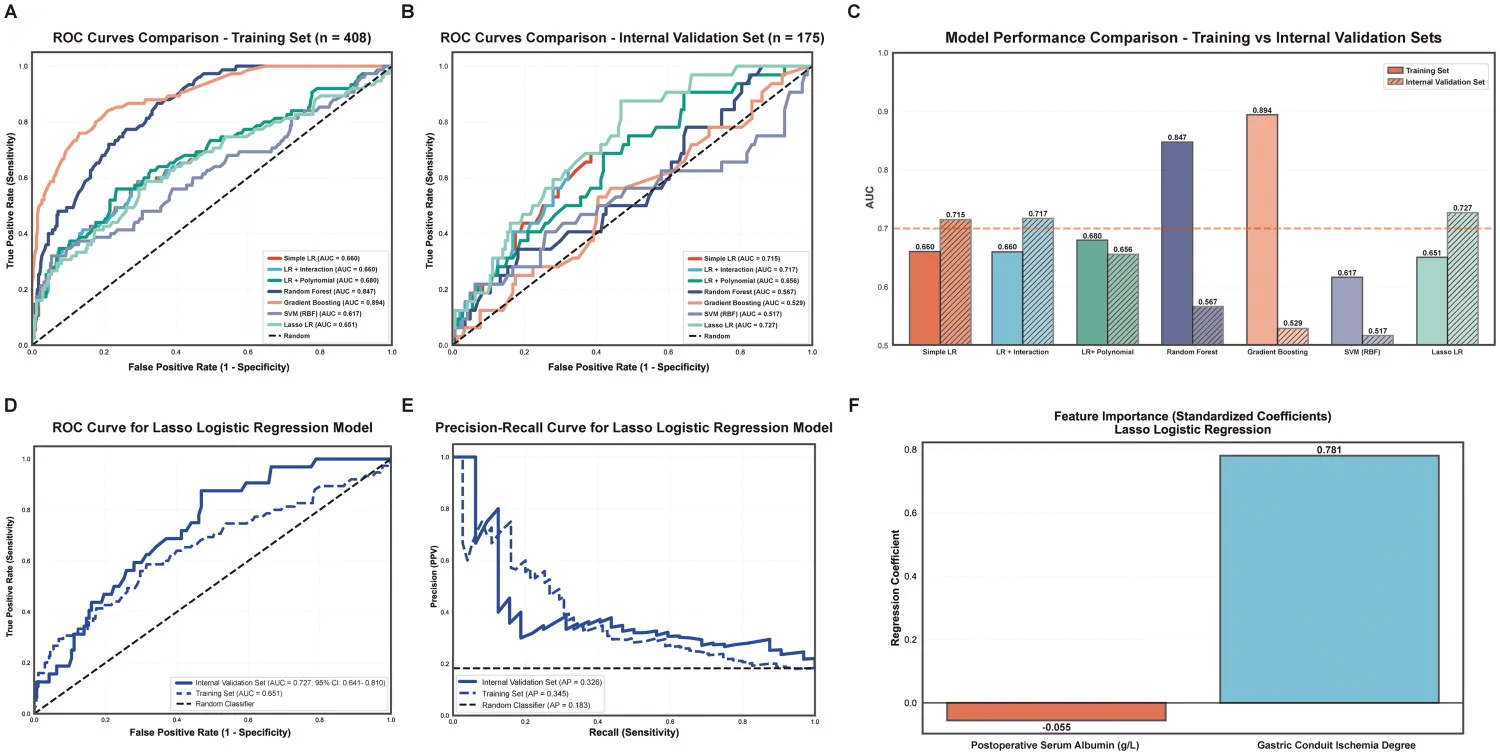
Predictive performance and feature importance of models for predicting AL after MIE. (A) ROC curves of seven different modeling approaches for predicting AL in the training set (*n* = 408). (B) ROC curves of the seven modeling approaches in the internal validation set (*n* = 175). (C) Comparison of model performance (AUC values) between the training and internal validation sets, demonstrating the excellent generalizability of the Lasso logistic regression model. (D) Dedicated ROC curves of the optimal Lasso logistic regression model in the training and internal validation sets. (E) PRC of the Lasso logistic regression model in the training and internal validation sets, used to evaluate its predictive stability for an imbalanced outcome. (F) Feature importance based on the standardized regression coefficients in the final Lasso logistic regression model. ROC, receiver operating characteristic; LR, logistic regression; Lasso, least absolute shrinkage and selection operator; AP, average precision.

The overall cohort of 583 patients was randomly divided into a training set (*n* = 408) and an internal validation set (*n* = 175) at a 7:3 ratio. Seven machine-learning and statistical models were developed and systematically compared for their ability to predict AL after MIE. ROC analysis showed that the Lasso LR model had the best discriminative performance in the internal validation set, with an AUC of 0.727 (95% CI: 0.641–0.810). In contrast, complex nonlinear models such as gradient boosting (AUC = 0.529) and random forest (AUC = 0.567) performed poorly in the internal validation set. The Lasso LR model performed slightly better in the internal validation set than in the training set (AUC = 0.651), suggesting good generalizability without clear evidence of overfitting. In contrast, the random forest (AUC = 0.847) and gradient boosting (AUC = 0.894) models performed well in the training set but showed substantial declines in the internal validation set, suggesting overfitting (Figure 5A–C). These findings suggest that Lasso LR, a linear model incorporating feature selection and regularization, was the optimal choice for prediction in this study (Figure 5D).

Because AL was an imbalanced outcome in this cohort, PRCs were also used for complementary evaluation. The Lasso LR model achieved an AP of 0.326 in the internal validation set and 0.345 in the training set, exceeding the AP of a random classifier (0.183), indicating that the model maintains reliable precision in predicting AL (Figure 5E). Following confirmation of model robustness, the relative contribution of each predictor was quantified by calculating standardized regression coefficients (Figure 5F). The results showed that the risk contribution of gastric conduit ischemia degree (coefficient = +0.781) was substantially greater than the protective effect of postoperative serum albumin (coefficient = −0.055).

### Development of a nomogram and digital prediction tools and validation of clinical utility

**Figure 6.**
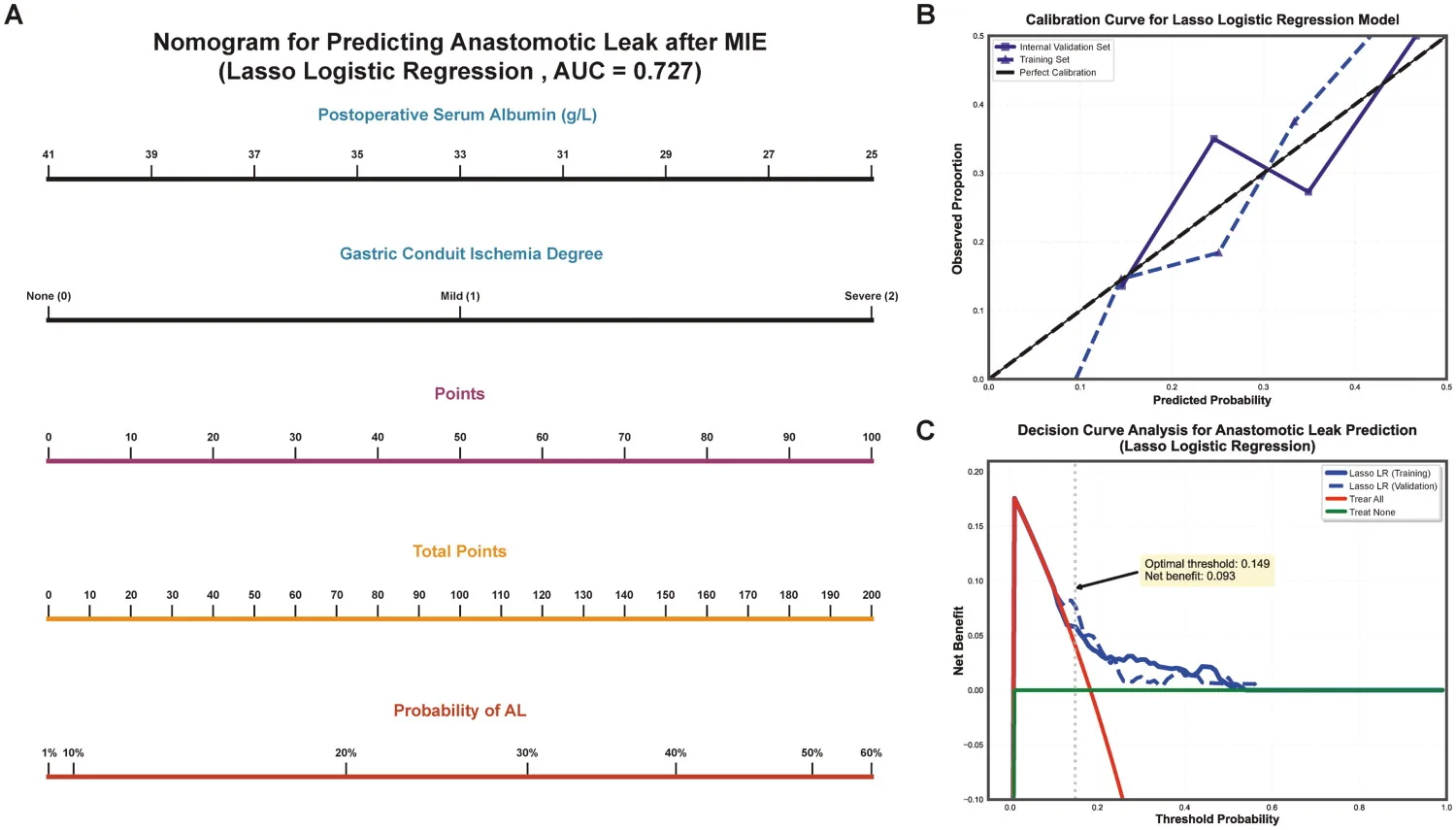
Development and clinical utility assessment of the nomogram for predicting AL after MIE. (A) Individualized AL risk prediction nomogram developed based on the optimal Lasso logistic regression model. (B) Calibration curves of the nomogram in the training and internal validation sets, demonstrating excellent agreement between predicted probabilities and observed outcomes. (C) DCA evaluating the net clinical benefit of the nomogram across a wide range of threshold probabilities. MIE, minimally invasive esophagectomy; AL, nastomotic leak; DCA, decision curve analysis; LR, logistic regression.

To translate the optimal model into a practical tool for rapid bedside assessment, a nomogram was developed to estimate the individual risk of AL based on postoperative serum albumin and gastric conduit ischemia degree (Figure 6A). Internal validation with calibration curves showed good agreement between predicted and observed AL probabilities (Figure 6B), without significant systematic bias. DCA further showed that use of the nomogram provided a positive net clinical benefit across a broad range of threshold probabilities (Figure 6C). At the risk probability threshold corresponding to the Youden index (14.9%), the model had a net benefit of 0.093. This finding suggests that the nomogram may help identify patients at increased risk while limiting unnecessary interventions among those at lower risk.

To improve the usability and accessibility of this predictive model in routine clinical practice, an interactive web-based risk assessment tool was also developed (Supplementary Material 2). Clinicians can enter the patient’s postoperative serum albumin level and gastric conduit ischemia degree, after which the system automatically calculates the predicted probability of AL. The introduction of these digital tools substantially simplifies the assessment process, enabling the predictive model to be integrated efficiently and seamlessly into routine bedside practice. Based on the individual predicted probabilities generated by the digital tools and the ROC curve derived from the internal validation set, two key probability thresholds were established to construct a clinical risk stratification framework. First, the optimal cutoff value determined by the Youden index was 14.9% (sensitivity 87.5%, specificity 53.1%), used to maximize risk identification. Second, to identify extremely high-risk patients, the lowest probability value corresponding to a specificity ≥ 90% was selected as the high-risk threshold, which was 30.1% (specificity 93.7%, sensitivity 18.8%). Accordingly, clinicians can directly use the AL probability calculated by the model or digital tools to stratify patients into three risk groups: low-risk (< 14.9%), medium-risk (≥ 14.9% – < 30.1%), and high-risk (≥ 30.1%). This stratification provides an objective data-driven foundation for implementing individualized stepwise postoperative monitoring strategies.

### Association of previously identified predictors with AL severity

To assess the association between AL severity and the previously identified predictors, the 107 patients who developed AL were stratified according to the ECCG classification. AL severity was distributed as follows: ECCG Type I, 46 patients (42.99%); Type II, 56 patients (52.34%); and Type III, 5 patients (4.67%) (Table S1).

Gastric conduit ischemic length and postoperative serum albumin levels were then compared among patients without AL (Non-AL group, *n* = 476) and patients with ECCG Type I, II, or III AL. The Kruskal–Wallis test demonstrated significant differences in both variables across the groups (Table S2). Descriptive inspection of the distributions suggested that postoperative serum albumin levels decreased progressively, whereas gastric conduit ischemic length increased progressively, with increasing ECCG severity (Figure S2A,B).

Spearman analysis demonstrated a significant inverse correlation between postoperative serum albumin level and ECCG type (*r* = −0.1652, 95% CI: −0.2454 to −0.0828; *p* < 0.0001), and a significant positive correlation between gastric conduit ischemic length and ECCG type (*r* = 0.2168, 95% CI: 0.1357–0.2951; *p* < 0.0001) (Figure S2C,D). These findings suggest that the extent of gastric conduit ischemia and postoperative serum albumin level were associated with AL severity and may have potential clinical utility in perioperative risk assessment. These variables may also provide objective parameters for assessing gastric conduit perfusion and optimizing nutritional support in patients undergoing esophagectomy.

## Discussion

In this prospective cohort study, greater gastric conduit ischemic length and lower postoperative serum albumin were independently associated with an increased risk of AL after MIE, supporting the proposed roles of local conduit perfusion and systemic healing capacity in anastomotic healing. Subsequently, these two variables were incorporated into a risk-stratification model, a nomogram, and interactive digital calculators for individualized early postoperative risk assessment. Through this approach, earlier identification of patients at increased risk of AL within the critical 72-hour postoperative period may be achieved. To our knowledge, this study is the first to enable individualized quantitative assessment of AL risk within the critical 72-hour window after surgery. The potential clinical value of this research lies in its departure from the traditional paradigm of passive diagnosis based on delayed symptoms or radiographic signs. By integrating local microcirculatory status with systemic nutritional status, this model facilitates earlier risk stratification and may help inform timely clinical evaluation.

The quality of anastomotic healing fundamentally depends on local tissue perfusion. In this study, gastric conduit ischemic length directly reflected the microcirculatory perfusion status of the anastomotic region. The threshold of ≥ 3.5 cm may be related to the vascular anatomy of the distal gastric conduit. After esophagectomy, the gastric conduit depends predominantly on the right gastroepiploic artery for blood supply, and the anastomotic site at the gastric fundus lies at the distal end of this vascular supply. An ischemic zone exceeding 3.5 cm may indicate that microthrombosis or vasospasm within this region exceeds the compensatory capacity of the tissues, preventing the formation of a stable physical barrier by collagen fibers during the critical period of 3–5 days postoperatively [24–26]. The innovation of this study lies in the transformation of blood supply assessment from traditional subjective judgment into objective, quantifiable metrics through early postoperative endoscopy, providing reproducible criteria for clinical evaluation.

Equally important as local blood supply is the adequacy of systemic nutritional substrates. Abnormal decreases in postoperative serum albumin reflect the systemic pathological state induced by surgical stress, arising from the systemic inflammatory response triggered by surgical trauma, which increases vascular permeability leading to albumin leakage, as well as accelerated protein catabolism due to hypermetabolism under stress conditions [27–29]. Reduced serum albumin can decrease plasma colloid osmotic pressure, promote fluid movement into the interstitial space, and contribute to edema around the anastomosis and surrounding tissues [30,31]. The cutoff value of < 31.75 g/L identified in this study suggests that when postoperative serum albumin falls below this critical point, the negative impact of systemic status on healing reaches a decompensatory threshold.

Exploratory severity-stratified analyses showed statistically significant but modest ordinal associations between ECCG type and the two predictors: postoperative serum albumin was inversely associated with ECCG type, whereas gastric conduit ischemic length was positively associated with ECCG type. These findings suggest that the two variables may also be related to AL severity. However, given the modest correlation coefficients and the small number of patients with Type III AL, these results should be interpreted cautiously and require confirmation in larger cohorts.

Notably, a potential synergistic interaction may exist between local ischemia and low postoperative serum albumin. This synergy manifests as a self-perpetuating vicious cycle: gastric conduit ischemia leads to inadequate tissue oxygen supply, while low serum albumin induces interstitial edema due to reduced colloid osmotic pressure, which generates mechanical pressure that compresses microvessels and increases the diffusion distance for oxygen, thereby exacerbating local microcirculatory compromise at both physical and biochemical levels [32]. This pathological process, in which ischemia leads to edema, which in turn aggravates ischemia, combined with impaired immune function and tissue repair capacity under hypoproteinemic conditions, creates an unfavorable environment for anastomotic healing. This underscores that anastomotic healing is influenced by both the local tissue microenvironment and systemic physiological status. Therefore, based on the combined warning model developed in this study, clinical intervention strategies must transcend unidimensional approaches and simultaneously address improvements in local blood supply and systemic support to effectively reduce the risk of AL.

Compared with previously published prediction models for postoperative AL, the present model has several distinctive features. Existing models have predominantly incorporated preoperative characteristics, comorbidities, operative variables, or general postoperative inflammatory markers [13,14]. Although useful for broad perioperative risk estimation, such models may not capture early local perfusion status of the gastric conduit, which is central to anastomotic healing. The present model combines quantitative early postoperative assessment of gastric conduit ischemic length with postoperative serum albumin, thereby incorporating indicators of both local perfusion and systemic nutritional status. This may enhance its clinical relevance for early bedside risk stratification and targeted intervention. Direct external comparisons with other published models are needed to determine whether this approach provides incremental predictive value in different clinical settings.

Compared with existing postoperative warning tools, the model developed in this study offers significant advantages in both timeliness and mechanistic insight. Traditional inflammatory markers, such as C-reactive protein (CRP), typically reflect a delayed response following tissue leakage or infection [33]. In contrast, gastric conduit ischemic length and postoperative serum albumin, the two factors captured in this study, are antecedent pathological factors that directly impair healing, enabling early warning prior to overt perforation. Furthermore, while intraoperative indocyanine green (ICG) angiography can only assess immediate blood flow at the time of surgery and cannot predict delayed microcirculatory impairment caused by edema or microthrombosis [34], early postoperative endoscopy at 72 h serves as a critical spatial and temporal extension of intraoperative assessment. It dynamically captures the actual evolution of anastomotic microcirculation under stress conditions, thereby providing robust evidence for individualized precision intervention during the critical time window for clinical management.

In this cohort, traditional risk factors such as anastomotic location and history of neoadjuvant therapy did not demonstrate significant associations. One possible explanation is the highly standardized minimally invasive anastomotic technique employed at our institution, which effectively neutralized the risk variations associated with anatomical location and preoperative treatment. In addition, PSM rigorously eliminated baseline confounding, allowing isolation of the core determinants of anastomotic outcome. These findings suggest that local blood supply and systemic nutritional status may be the critical factors governing anastomotic healing. Rather than negating the importance of traditional risk factors, this observation highlights that in centers where surgical techniques are highly standardized, these two core elements become the ultimate rate-limiting steps in determining anastomotic healing quality.

In terms of clinical translation, the risk stratification model developed in this study, together with the precise predictive tool based on Lasso logistic regression, constitutes a decision-support system that spans from rapid population-level screening to individualized quantitative assessment. Through this model, the effects of the two core predictors, postoperative serum albumin (negative coefficient, −0.055) and gastric conduit ischemia degree (positive coefficient, +0.781), were visualized in a nomogram, and a portable spreadsheet-based risk calculator as well as an interactive web-based risk assessment tool were developed. These digital tools enable real-time bedside prediction: within 72 h after esophageal cancer surgery, entry of these two objective parameters allows calculation of the predicted probability of AL. Their use is therefore contingent on the timely availability and reliable measurement of both predictors. This synergistic model of rapid screening combined with digital precision measurement enhances the feasibility and accuracy of clinical assessment, representing an important pathway toward individualized precision medicine after esophagectomy.

The predicted probabilities generated by the model may provide a basis for future prospective evaluation of risk-adapted postoperative surveillance strategies. At present, the probability thresholds of 14.9% and 30.1% should be regarded as exploratory, data-derived cutoffs rather than established clinical intervention thresholds. Patients with higher predicted risk may warrant closer clinical assessment and consideration of additional laboratory, radiographic, or endoscopic evaluation according to their overall clinical condition. However, the present observational study does not establish that prolonged fasting, extended antibiotic treatment, repeated endoscopy, or other model-guided interventions improve clinical outcomes. Management decisions should therefore not be based on the model alone. Prospective clinical impact studies are required to determine whether model-assisted surveillance can reduce AL-related morbidity or improve patient outcomes.

Beyond its immediate clinical utility, future mechanistic studies will further strengthen this model’s translational foundation. The ischemic threshold (≥ 3.5 cm) may reflect microvascular pathology; thus, future research should correlate this endoscopic finding with tissue-level markers such as microvessel density, Hypoxia‑inducible factor‑1α (HIF-1α), and vascular endothelial growth factor (VEGF). This could validate the cutoff’s biological plausibility and uncover therapeutic targets for promoting neovascularization. Similarly, the local impact of postoperative hypoalbuminemia, specifically its role in promoting interstitial edema and suppressing collagen synthesis, warrants histological investigation. Integrating these molecular endpoints will evolve this predictive model from a statistical tool into a mechanistically grounded framework for precision medicine.

This study offers notable strengths in clinical design and methodology. The prospective cohort design and standardized endoscopic assessment protocol performed within 72 h postoperatively not only ensured the objectivity of the observed indicators but also secured a critical window for early warning before the onset of severe clinical symptoms of AL. Furthermore, in terms of model selection strategy, this study demonstrated that the regularized Lasso logistic regression model outperformed complex black-box machine learning algorithms given the current sample size, and the use of PRCs confirmed its predictive reliability in the context of imbalanced data.

Although the model performed well in the training and internal validation cohorts, its clinical use should be interpreted cautiously. First, this single-center prospective cohort study included internal validation only; therefore, the model’s generalizability to independent populations, particularly those from different geographic regions and ethnic backgrounds, remains uncertain. Prospective multicenter external validation is required before routine clinical use. Second, although software-assisted assessment was used to evaluate the seven endoscopic features, the absence of image-enhanced endoscopy, such as narrow-band imaging (NBI), may have introduced subjectivity into endoscopic interpretation. Third, the high-risk subgroup in the simplified risk-stratification model was small (*n* = 19), resulting in imprecise estimates and wide confidence intervals. The AL incidence and model performance in this subgroup therefore require confirmation in larger cohorts. Finally, despite standardized protocols and software-assisted measurements, interobserver variability may persist in assessing gastric conduit ischemia, particularly when delineating ischemic boundaries, selecting representative images, and interpreting mucosal color and viability. Institution-specific differences in surgical expertise, operative and anastomotic techniques, perioperative nutritional support, postoperative surveillance, and early endoscopic assessment may further influence baseline leakage risk and the effects of individual predictors, thereby affecting model transportability.

These considerations also define the conditions for reliable implementation. Model implementation requires valid measurements of gastric conduit ischemic length and postoperative serum albumin within 72 h of surgery. Completeness and measurement quality should be verified before calculation; if either predictor is missing or unreliable, the model should not be used without a validated imputation strategy. External adoption therefore requires standardized endoscopic and laboratory assessment, while clinical judgment should prevail when reliable inputs are unavailable.

Future studies should prioritize prospective multicenter external validation in geographically and ethnically diverse populations, together with interinstitutional reproducibility studies of gastric conduit ischemia assessment. These studies should evaluate model calibration, clinical utility, and the need for local recalibration or updating. Broader implementation will also require standardized assessment criteria, structured operator training, and further refinement of the accompanying digital platform.

In conclusion, gastric conduit ischemic length ≥ 3.5 cm and lower postoperative serum albumin were independently associated with subsequent AL after MIE. Based on these variables, a simplified risk-stratification system, a nomogram, and digital calculators were developed using endoscopic and biochemical information obtained within the first 72 postoperative hours. Exploratory analyses also suggested that the two variables were associated with ECCG-defined AL severity, although these findings require cautious interpretation because of the small number of patients with Type III AL. The proposed tools may support early postoperative risk stratification and help identify patients who require closer clinical evaluation. However, their predictive performance, transportability, and clinical impact require independent external validation and prospective impact assessment before they can be used to guide routine clinical management.

## Supplementary Material

Supplemental Material

Fig_S1.tif

Supplementary material 2_2.docx

Supplementary_material_1_TRIPODAI_checklist.docx

Fig_S2.tif

## Data Availability

The study data, protocol and the analytical code used for data cleaning, feature engineering, model building, and evaluation are available upon reasonable request to the corresponding author. Requests will be assessed for scientific rigor before approval. Data will be anonymized and securely transferred.

## Open Scholarship

This article has earned the Center for Open Science badges for Preregistered. The materials are openly accessible at ChiCTR2100054550. Registered December 19, 2021. https://www.chictr.org.cn/showproj.html?proj=144621.
