# Supplementary material for: Development and internal validation of a prediction model for early anastomotic leak after minimally invasive esophagectomy: a prospective cohort study
Source: Ann Med. 2026 Sep 12;58(1):2726636. doi: 10.1080/07853890.2026.2726636 (PMC13573515; doi:10.1080/07853890.2026.2726636)
Supplement: Supplementary file 2 [file IANN_A_2726636_SM0509.docx]

| **AL Risk Prediction Calculator** | | | |
| --- | --- | --- | --- |
| Lasso Logistic Regression Model (AUC = 0.727, 95% CI: 0.641-0.810) | | | |
|  |  |  |  |
| **Input Parameters** |  |  |  |
| Post-op Albumin (g/L): | 35 | Range: 20-50 |  |
| Ischemia Degree: | 0 | 0=None, 1=Mild, 2=Severe |  |
|  |  |  |  |
| Instructions: |  |  |  |
| 1. Enter albumin value in B5 (20-50) |  |  |  |
| 2. Enter ischemia degree in B6 (0/1/2) |  |  |  |
| 3. Results will be automatically calculated |  |  |  |
|  |  |  |  |
| **Prediction Results** |  |  |  |
| AL Risk Probability: | **12.57** | % |  |
| Risk Category: | **Low Risk** |  |  |
| Clinical Recommendation: | **Routine postoperative management** |  |  |
|  |  |  |  |
| **Model Information** |  |  |  |
| Model Type: | **Lasso Logistic Regression (LR with L1)** |  |  |
| AUC: | **0.7268 (95% CI: 0.6408-0.8104)** |  |  |
| Optimal Threshold: | **0.149 (14.9%)** |  |  |
| Sensitivity: | **0.875 (87.5%)** |  |  |
| Specificity: | **0.531 (53.1%)** |  |  |
| PPV: | **0.295 (29.5%)** |  |  |
| NPV: | **0.950 (95.0%)** |  |  |
| Albumin Coefficient: | **-0.0554 (negative)** |  |  |
| Ischemia Coefficient: | **0.7806 (positive)** |  |  |
| Training Sample Size: | **408 patients** |  |  |
| Testing Sample Size: | **175 patients** |  |  |
