# Supplementary material for: Development and internal validation of a prediction model for early anastomotic leak after minimally invasive esophagectomy: a prospective cohort study
Source: Ann Med. 2026 Sep 12;58(1):2726636. doi: 10.1080/07853890.2026.2726636 (PMC13573515; doi:10.1080/07853890.2026.2726636)
Supplement: Supplementary file 3 [file IANN_A_2726636_SM7535.docx]

**Supplementary Tables**

| **Table S1. Distribution of ECCG AL Severity Types (n=107)** | | |
| --- | --- | --- |
| **ECCG AL severity** | **n** | **Proportion (%)** |
| Type I | 46 | 42.99 |
| Type II | 56 | 52.34 |
| Type III | 5 | 4.67 |
| Total | 107 | 100.00 |
| AL, anastomotic leakage; ECCG, Esophagectomy Complications Consensus Group. | | |

| **Table S2. Comparison of clinical parameters among Non-AL and ECCG Type I-III AL patients.** | | | | | |
| --- | --- | --- | --- | --- | --- |
| **Variable** | **Non-AL**  **(n = 476)** | **ECCG Type I**  **(n = 46)** | **ECCG Type** **II**  **(n = 56)** | **ECCG Type** **III**  **(n = 5)** | **P Value** |
| Postoperative serum albumin | 32.4 [30.03, 34.65] | 30.65 [28.98, 33.48] | 30.20 [28.25, 33.20] | 31.7 [28.05, 35.45] | 0.0009 |
| Gastric conduit ischemic length | 0.00 [0.00, 0.00] | 0.00 [0.00, 0.50] | 0.00 [0.00, 3.00] | 1.00 [0.00, 4.50] | < 0.0001 |
| AL, anastomotic leak; ECCG, Esophagectomy Complications Consensus Group. | | | | | |

Fig. S1 Patients in the AL and non-AL groups were matched using PSM.

(A) Scatter plot of propensity score distribution. It is divided vertically into unmatched treated units, matched treated units, matched control units, and unmatched control units. (B) Forest plot of ASMD for covariates. The vertical axis lists various covariates. The horizontal axis represents the ASMD. Open circles represent the full unmatched sample (all), and solid circles represent the matched sample (matched). PSM, Propensity score matching. ASMD, Absolute standardized mean difference.

**Fig. S2 Correlation between postoperative serum albumin, gastric conduit ischemic length, and AL severity in esophagectomy patients.**

(A) Postoperative serum albumin levels across different groups. (B) Gastric conduit ischemic length across different groups. (C) Correlation analysis between postoperative serum albumin and ECCG type. (D) Correlation analysis between gastric conduit ischemic length and ECCG type.

Data are presented as median with interquartile range. Comparisons were performed using Kruskal-Wallis test (A, B) or Spearman correlation test (C, D), Mann-Whitney U test was used for pairwise comparisons between each group of A and B. *P < 0.05; **P < 0.01; ****P < 0.0001. AL, Anastomotic leak; ECCG, Esophagectomy Complications Consensus Group.
